# Mechanisms of Methylparaben Adsorption onto Activated Carbons: Removal Tests Supported by a Calorimetric Study of the Adsorbent–Adsorbate Interactions

**DOI:** 10.3390/molecules24030413

**Published:** 2019-01-23

**Authors:** Valentina Bernal, Liliana Giraldo, Juan Carlos Moreno-Piraján, Marco Balsamo, Alessandro Erto

**Affiliations:** 1 Departamento de Química, Universidad Nacional de Colombia, Bogotá 11001, Colombia; vbernalf@unal.edu.co (V.B.); lgiraldogu@unal.edu.co (L.G.); 2 Departamento de Química, Universidad de los Andes, Bogotá 111711, Colombia; 3 Departamento di Ingegneria Chimica, dei Materiali e della Produzione Industriale, Università degli Studi di Napoli Federico II, 80121-80147 Napoli, Italy; marco.balsamo@unina.it (M.B.); aleserto@unina.it (A.E.)

**Keywords:** adsorption mechanisms, activated carbon, methylparaben, calorimetry, immersion enthalpy, surface functional groups

## Abstract

In this study, the mechanisms of methylparaben adsorption onto activated carbon (AC) are elucidated starting from equilibrium and thermodynamic data. Adsorption tests are carried out on three ACs with different surface chemistry, in different pH and ionic strength aqueous solutions. Experimental results show that the methylparaben adsorption capacity is slightly affected by pH changes, while it is significantly reduced in the presence of high ionic strength. In particular, methylparaben adsorption is directly dependent on the micropore volume of the ACs and the π- stacking interactions, the latter representing the main interaction mechanism of methylparaben adsorption from liquid phase. The equilibrium adsorption data are complemented with novel calorimetric data that allow calculation of the enthalpy change associated with the interactions between solvent-adsorbent, adsorbent-adsorbate and the contribution of the ester functional group (in the methylparaben structure) to the adsorbate–adsorbent interactions, in different pH and ionic strength conditions. It was determined that the interaction enthalpy of methylparaben-AC in water increases (absolute value) slightly with the basicity of the activated carbons, due to the formation of interactions with π- electrons and basic functional groups of ACs. The contribution of the ester group to the adsorbate-adsorbent interactions occurs only in the presence of phenol groups on AC by the formation of Brønsted–Lowry acid–base interactions.

## 1. Introduction

Methylparaben is a preservative widely used in the cosmetics, pharmaceutical and food industries due to its antibacterial and antifungal properties. Currently, it is considered among the so-called “emerging pollutants”, due to its increasing incidence in surface water, drinking water and soil [1]. Moreover, it is suspected to be potentially dangerous to human health due to the disruptor effect that this molecule has on the endocrine system and its relationship with the growth of cells associated with breast cancer [2].

Although this compound is biodegradable, this degradation mechanism is insufficient to remove the great amount constantly discharged in water and then accumulating in the ecosystems. In the literature, bioaccumulation of parabens in aquatic organisms is reported [3]. Zgoła-Grześkowiak et al. [4] indicate that methylparaben and propylparaben are among the most widely detected parabens in freshwater in South Wales rivers, Japanese urban streams, Swiss rivers and Antarctic seawater. In general, the quantification of these molecules in aqueous matrices is carried out with high-performance liquid chromatography or gas chromatography, whereas solid phase extraction (SPE) and dispersive liquid–liquid microextraction are used after filtration of water to clean and concentrate the sample. Detection systems included ultraviolet, electrochemical, fluorimetric, photoionization, and mass spectrometry [4].

Conventional wastewater treatment facilities typically include biological systems using the activated sludge process, whereas advanced treatment can also include tertiary treatment processes, such as reverse osmosis, ozonation and advanced oxidation technologies (e.g., with TiO_2,_ ferrate (VI), H_2_O_2_). However, none of the wide range of water treatment processes currently available has been specifically designed to remove pharmaceuticals. Nonetheless, granular activated carbon (GAC) and powdered activated carbon (PAC) are increasingly adopted in drinking water treatment to remove pesticides as well as some pharmaceuticals, hence their use can also be proficiently extended to the treatment of wastewater [5,6,7].

Adsorption is an interesting alternative to other tertiary water treatments such as chlorination, advanced oxidation and ozonation when the molecules are reactive (as methylparaben) and can form toxic compounds, such as dioxins, organochlorine compounds and chloroamines upon oxidation [8,9,10]. Adsorption processes strictly depends on physical (e.g., surface area and pore volume of the adsorbent, molecular size of the adsorbate, etc.) and chemical factors (e.g., acidity or basicity of the adsorbent, pKa of the adsorbate, etc.), and on the specific couple adsorbent–adsorbate. In order to reach an adequate knowledge of the main aspects of the process applied to the removal of a specific pollutant, a dedicated study performed in different experimental conditions, which modify the physico-chemical characteristics of the system, is of utmost importance, so as to obtain useful indications about the removal mechanisms and precise information about the most appropriate process operating parameters [5,11,12,13].

In the literature, the use of activated carbon (AC) as adsorbent in tertiary water treatments is widely adopted due to its recognized characteristics, such as high surface area and tunable porosity [8,14,15,16,17]. In addition, the different functional groups present on its surface can facilitate the interactions with molecules of different chemical nature. In the specific case of aromatic compounds such as methylparaben, the surface chemistry plays a relevant role and is directly correlated with the adsorption capacity [18]. For methylparaben, it can be hypothesized that the adsorbate–adsorbent interactions are similar to those presented by phenol, since this latter group is part of the methylparaben molecular structure. Therefore, it is expected that the adsorption capacity decreases in the presence of a high concentration of oxygenated groups (acids) on the activated carbon and increases with the basicity of the adsorbent. Simultaneously, the adsorbed amount of methylparaben should decrease along with a pH decrease and an increase in ionic strength [1,13,18,19,20,21]. However, dealing with adsorption, the analysis of the particular adsorbent–adsorbate couple is crucial as each adsorbate has its molecular properties and unpredictable results can arise. The literature reports the analysis of methylparaben adsorption on activated carbons or other adsorbents with phenol groups on the surface [1]; however, the analysis of the specific adsorbate–adsorbent interactions, which play a central role in the adsorption performances, has been little discussed.

In this work, the adsorption on methylparaben on three activated carbons with different physicochemical properties was investigated. Equilibrium data were evaluated at different pH and ionic strengths, in order to account for their effect on the adsorbate–adsorbent interactions and, consequently, on the adsorption capacity. To this aim, adsorption isotherm results were correlated to calorimetric data (i.e., immersion enthalpies), which allowed evaluation of the adsorbate–adsorbent-solvent interactions, where the adsorbate–adsorbent interactions were calculated with the Hess law. Additionally, immersion calorimetry tests in phenol solutions were performed to compare phenol-activated carbon and methylparaben-activated carbon interactions, so as to assess the specific contribution of the ester group, which differentiate methylparaben structure from that of the phenols, to the adsorbate–adsorbent interactions. The final goal of the work was to elucidate the adsorption mechanisms of methylparaben onto activated carbon based on a cross-analysis of equilibrium data, adsorbate–adsorbent interactions and textural properties of the adsorbents.

## 2. Results and Discussion

### 2.1. Activated Carbons Characterization

Nitrogen adsorption data at 77 K allows obtaining quantitative information about the adsorbent textural properties (i.e., micropore volume, total pore volume, apparent surface area) when applying mathematical models such as Brunauer–Emmett–Teller (BET), Dubinin–Astakhov (DA) and Quenched Solid Density Functional Theory (QSDFT) to raw adsorption data. These properties are relevant in the adsorption processes with porous materials because the adsorption capacity of different pollutants can be highly influenced e.g., for steric hindrance phenomena and, more generically, for the number of active sites effectively available for the adsorbate. In general, in the adsorption of organic molecules with small molecular size, the adsorption capacity is expected to increase when greater surface area and micropore volume are available [22].

In this work, three activated carbons were tested as adsorbents, a commercial activated carbon (CB) and two activated carbons (CB1073 and CB1173) obtained by CB through a same thermal treatment but carried out at different temperature, i.e., 1073 and 1173 K, respectively. The choice of the temperatures of treatment was made on the basis of the thermal instability of the functional groups possibly present on the activated carbon surface and aiming at obtaining two samples with different textural and chemical properties, as reported in a previous work of our research group [23].

**Figure 1:**
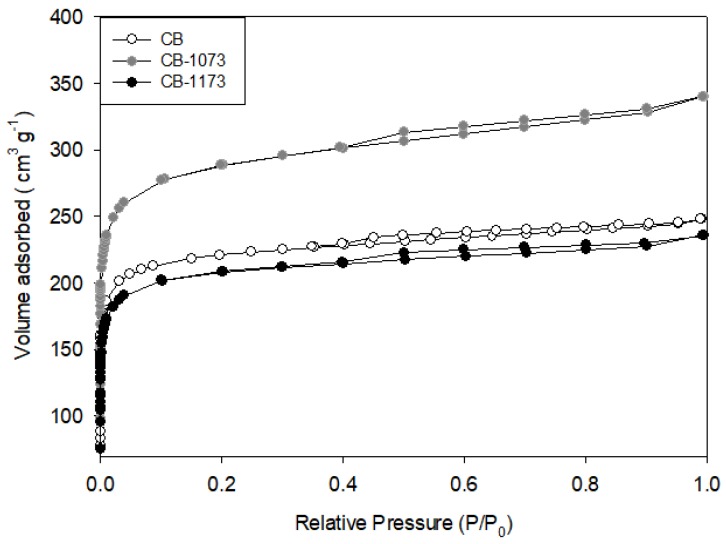
Nitrogen adsorption isotherms on CB, CB1073 and CB1173 activated carbons at 77 K.

The main textural characteristics of the activated carbons CB, CB1073, CB1173, as obtained from the nitrogen adsorption isotherms at 77 K (Figure 1), are presented in Table 1.

It can be observed that the activated carbons are prevalently microporous due to the small hysteresis loop formed in the desorption branch; this indicates a small capillary condensation and, therefore, a lower amount of mesopores.

**Figure 2:**
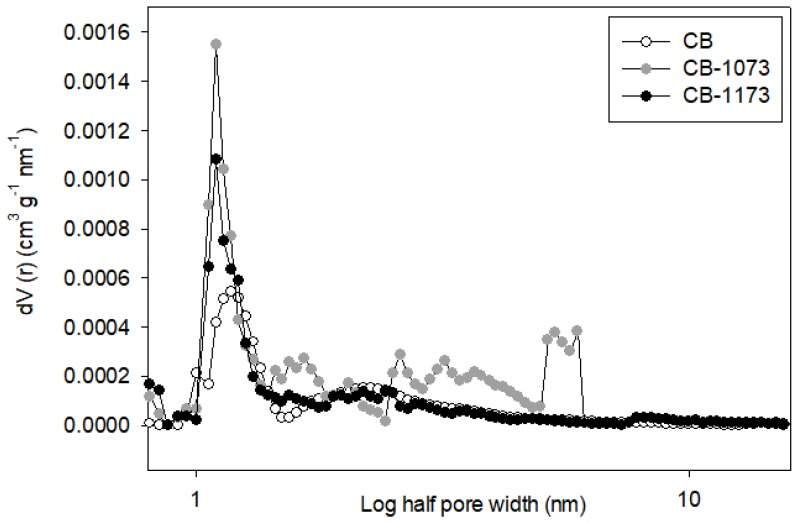
Pore size distribution of CB, CB1073, CB1173 activated carbons (methods: slit-cylinder pores, QSDFT adsorption branch and nitrogen isotherms).

The total volume and the pore sizes distribution of CB, CB1073 and CB1173 activated carbons were obtained by the QSDFT method assuming the pores as slit-cylindrical. Figure 2 shows the pore sizes distributions of the three investigated activated carbons.

From Figure 2, it can be confirmed the prevailing presence of micropores, mainly in the 1.0–1.5 nm region, for all the activated carbon samples, while for CB1073 activated carbon a small contribution of mesopores can be also detected.

The retrieved textural results can be ascribed to the effects of thermal treatments exerted on the textural properties of CB activated carbon. Indeed, from the data reported in Table 1, it can be observed that the increase in the temperature of thermal treatment above 1073 K is detrimental for textural properties (i.e., surface area and pore volume), while the treatment at 1073 K determines a significant increase in the values of these parameters with respect to the raw material (i.e., CB). For CB1173, it can be hypothesized that the worsening of the textural properties is associated to a pore;s blockage caused by the condensation products originated during the thermal treatment [22]. In addition, the rise in temperature up to 1173 K likely caused the collapse of carbonaceous structures, as testified by the significant decrease of the total and micropore volumes (Table 1). Marsh et al. [24] indicated that low treatment temperatures favour the formation of microporosity as is the case of CB1073 activated carbon, and the increase of the contribution of these pores favours the adsorption of nitrogen, which in turn testifies to an improvement in the textural parameters of this sample.

Table 2 shows the results of the chemical characterization of CB, CB1073 and CB1173 activated carbons.

According to Table 2, CB sample has a high amount of phenol groups because the coconut shell is constituted by lignin, cellulose and hemicellulose, all polymers having an abundant content of this group [25]. The thermal treatments at 1073 and 1173 K generated a decrease of phenol groups concentration because their temperature of thermal stability (623–923 K) was overpassed [26,27]. However, the amount of carboxylic acids increased despite the fact that the temperature of their thermal stability is in the range 523–623 K. This behaviour can be attributed to the reaction of free radicals formed in the reduction of oxygenated groups and carbon dioxide during the treatment [28]. For the same reasons, it is assumed that the lactones have a similar trend to carboxylic acids.

Simultaneously, the increase in the temperature of the thermal treatment favoured the increase in the total basicity of the activated carbons due to the formation of π electrons during the rearrangement of the graphene layers [29]. Likewise, the basic character of the thermal treated adsorbents are reflected by the pH of point of zero charge (pH_PZC_) values, which resulted equal to 11.1 and 8.90 for CB1073 and CB1173, respectively.

### 2.2. Methylparaben Adsorption onto Activated Carbon

It is well known that adsorption phenomena are significantly influenced by the properties of the activated carbon, which were reported in the previous section. However, it is equally demonstrated that some aspects related to the physicochemical properties of the adsorbate can be directly correlated with its adsorption capacity on a given adsorbent, taking into account that variables such as pH and ionic strength can modify the structure and the chemical properties of the solute. Table 3 shows some physicochemical properties of methylparaben (Methyl 4-hydroxybenzoate), referred to different solvent solutions.

Methylparaben is poorly soluble in water and HCl solutions, its solubility increases in basic pH solutions, so it is expected that the adsorbate–solvent interactions are weak at acid pH and increase along with pH. The addition of NaCl to the medium decreases the solubility of methylparaben by a salting out effect, disfavouring the interactions between the molecule and water [31]. On the other hand, the pKa slightly varies with the change of ionic strength. The data reported in Table 3 correspond to the pKa values corrected with the Debye–Huckel equation. In water, pKa is around 8.2 and the fraction of methylparaben molecules ionized is 50%, thus possibly modifying the type of interactions that are formed in the adsorption system, which also depend on the pH at the point of zero charge of the adsorbent.

Due to the methylparaben molecular dimensions (Table 3) and the pore size distribution of the activated carbons used in the experimental runs (Figure 2), there are no steric hindrance restrictions for methylparaben molecule accessibility in the sorbents pore network, since the smallest pore diameter (also corresponding to the greatest contribution to the total pore volume) is in the range between 1 and 1.5 nm (the contribution of pores smaller than 1 nm is practically negligible, as it is evident from Figure 2), dimensions being greater than the sizes of the adsorbate.

**Figure 3:**
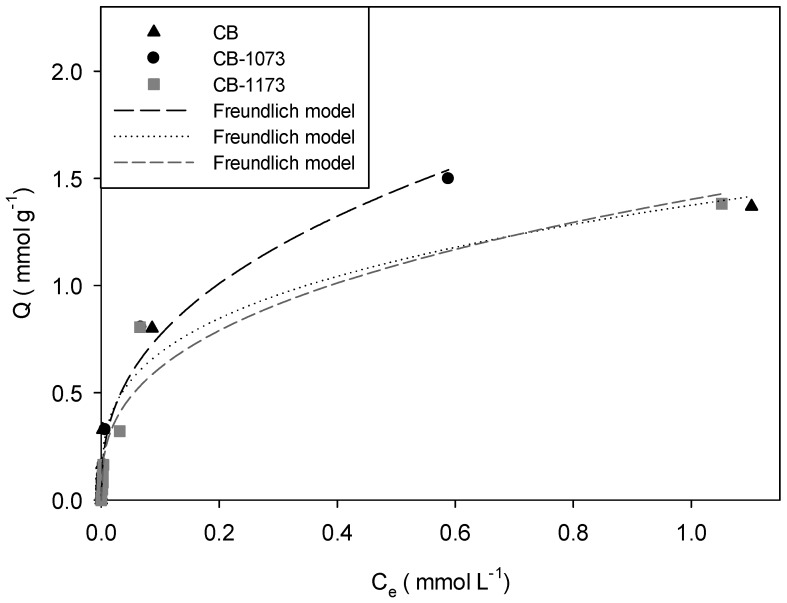
Adsorption isotherm of methylparaben on CB, CB1073 and CB1173 activated carbons in HCl 1 × 10^−2^ M solution at 293 K (Equilibrium pH: 1.18 ± 0.01).

**Figure 4:**
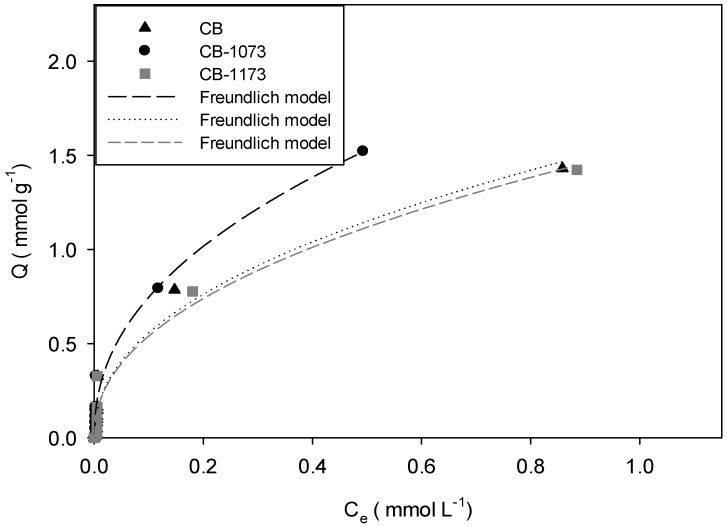
Adsorption isotherm of methylparaben on CB, CB1073 and CB1173 activated carbons in water at 293 K (Equilibrium pH: 7.75 ± 0.07).

**Figure 5:**
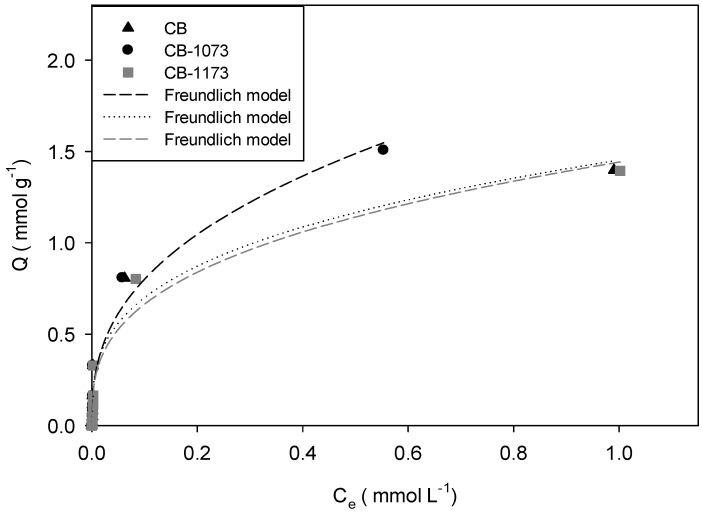
Adsorption isotherm of methylparaben on CB, CB1073 and CB1173 activated carbons in NaOH 1 × 10^−3^ M solution at 293 K (Equilibrium pH for CB and CB1173: 7.78 ± 0.02, Equilibrium pH for CB1073: 10 ± 0.03).

**Figure 6:**
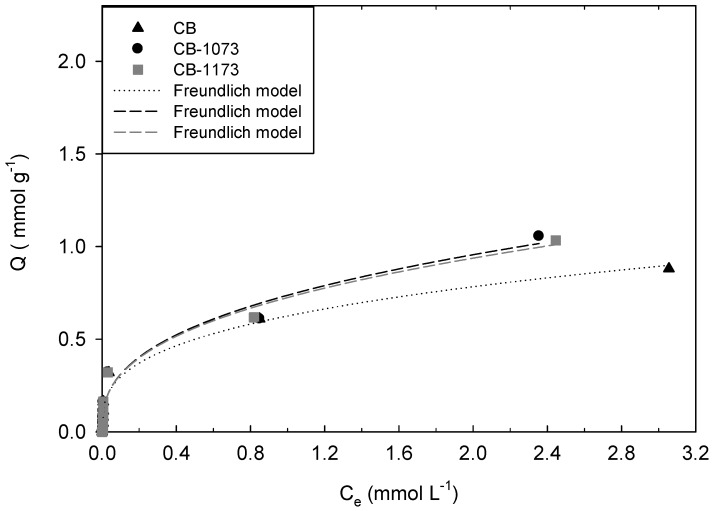
Adsorption isotherm of methylparaben on CB, CB1073 and CB1173 activated carbons in NaCl 1 × 10^−2^ M solution at 293 K (Equilibrium pH: 7.38 ± 0.35).

Figure 3, Figure 4, Figure 5 and Figure 6 show the adsorption isotherms of methylparaben at 293 K on the CB, CB1073 and CB1173 activated carbons in HCl solution, water, NaOH solution and NaCl solution 0.01M, respectively.

From Figure 3, Figure 4, Figure 5 and Figure 6, it can be noted that in all the tested experimental conditions, when equilibrium concentration is low (i.e., <0.1 mmol L^−1^) the differences in the amount adsorbed of methylparaben on the three activated carbons are minimal, while for higher equilibrium concentration, CB1073 showed the highest performances and CB and CB-1173 had a similar behaviour, except when ionic strength changes. In fact, in the latter case (Figure 6) the thermal treated samples showed the best performances and was almost similar. In order to analyse in detail the retrieved results, further investigations are needed and a modelling analysis might help.

Experimental data were modelled according to the commonly adopted models for organic compounds adsorption and retrievable from the pertinent literature. In Figure 3, Figure 4, Figure 5 and Figure 6, the model curves were also reported. It was found that the methylparaben adsorption on CB, CB1073 and CB1173 activated carbons can be adequately described by the Freundlich model, regardless of the change in the operating parameters used during the tests (i.e., pH and ionic strength). This likely indicates that the process take places by the formation of multilayers with non-homogeneous distributions of the adsorption interaction energy. According to this model, the active sites with the highest binding energy are occupied first, then the energy decreases exponentially. Equation (1) indicates that the adsorbed amount of methylparaben depends on the constant of the model, the equilibrium concentration of the adsorbate and a factor *n* associated with the heterogeneity of the adsorbent [32].

(1) $$Q=K_{F}{\left(C_{e}\right)}^{\frac{1}{n}}$$

From the statistical thermodynamics point of view, the value of the heterogeneity factor (*n*) depends on the coordination number of the adsorbate, the adsorbate–adsorbate interactions, the Boltzmann’s constant and the Avogadro’s number. Therefore, the heterogeneity of the systems depends on the binding energy between active sites and adsorbate and on the possible occurrence of lateral interactions [33]. Indeed, values of *n* that tend to 0 indicate physisorption while values close to 1 indicate chemisorption or cooperative adsorption.

In Table 4 the parameters of the Freundlich model were reported, as derived from the regression analyses of the experimental data (Figure 3, Figure 4, Figure 5 and Figure 6).

According to what was previously indicated, the adsorption of methylparaben on the activated carbons can be classified as mainly physisorption since the values of the parameter *n* are around 0.4, even if a contribution of active sites with different energy can be envisaged, as discussed in the following.

Once the best fitting model was assessed, it is possible to carry out a more precise and meaningful comparison among the methylparaben adsorbed amount on CB, CB1073 and CB1173 activated carbons, so to address the effect of the investigated operating parameters (i.e., pH and ionic strength). Indeed, in order to compare the performances of the three activated carbons, the adsorption capacity should be calculated at the same equilibrium concentration, retrievable from the model application using the model parameters reported in Table 4. To this aim, a concentration of 0.5 mmol L^−1^ was taken as equilibrium concentration (*C_e_)* and the corresponding amount of methylparaben adsorbed (*Q_0.5_*) was calculated for the different investigated conditions, as reported in Table 5.

The retrieved methylparaben adsorption capacity of CB and CB1173 activated carbons in the four different solutions resulted almost similar; this can be primarily attributed to their similar physicochemical characteristics, in particular in terms of BET surface area (S_BET_ CB = 864 m^2^ g^−1^; S_BET_ CB11173 = 814 m^2^ g^−1^) and micropore volume (V_m_ CB= 0.35 cm^3^ g^−1^; V_m_ CB1173 = 0.34 cm^3^ g^−1^). On the contrary, CB1073 showed the highest surface area and micropore volume, which resulted in the best performances in methylparaben adsorption.

However, a finer analysis can be carried out, also taking into account the chemical properties of both the adsorbents and the adsorbate. For all the investigated activated carbons, the ranking of adsorption capacity between the solution at different pH resulted the same (H_2_O ≅ NaOH > HCl), even if the differences are small. Adsorption in NaCl solution was confirmed to be the lowest by far. Indeed, the adsorption of methylparaben on CB, CB1073 and CB1173 activated carbons should be analysed also in light of the correlation between the chemical properties of the adsorbate–adsorbent system (which depend on the pKa of the adsorbate and on the pH_pzc_ of the adsorbent) and the experimental conditions adopted in this study. Table 6 shows the initial and final (i.e., at equilibrium) pH and the percentage of negatively ionized methylparaben in the adsorption solutions. The results were obtained using the Henderson–Hasselbach equation, the initial pH and the equilibrium pH data, as well as the pKa reported in Table 3.

From Table 6, it can be observed that, for each activated carbon, the % of ionization at equilibrium increases in the range HCl < H_2_O < NaOH, hence the ranking in adsorption capacity seems not to be significantly influenced by the chemical form of methylparaben in solution. In fact, the best adsorption performances were observed for CB1073 in water (% ionization = 29.9) and when the ionization percentage is the highest and almost complete (i.e., at basic pH), methylparaben adsorption capacity was very slightly affected. Moreover, for data obtained in NaCl solution, the ionization was comparable with HCl solution but the adsorption capacity was significantly lower.

The effect of ionization seems to be rather correlated to the pH_pzc_. When the former increases and the equilibrium pH is higher than pH_pzc_ (e.g., for CB activated carbon in basic conditions) an additional repulsive effect can arise between the negative dissociated methylparaben and the negatively charged adsorbent surface, hence potentially decreasing the adsorption capacity. However, except for the indicated case, the ionization percentage resulted always small and the effect exerted was small too. For CB1173, the higher percentage of dissociation (negative effect) and the higher pH_pzc_ (positive effect) seem to be counterbalanced, hence explaining its similar behaviour to CB. On contrary, the repulsive effect is never present on CB1073, being its pH_pzc_ always higher than equilibrium pH, and it resulted the most performing adsorbent. Further indications can be retrieved by comparative analyses of the data reported in Table 5 and Table 6. As an example, methylparaben adsorption at acid pH is favoured on CB1073 (Q_0.5_ CB1073 = 1.45 mmol g^−1^), followed by CB (Q_0.5_ CB1073 = 1.12 mmol g^−1^) and ending with CB1173 (Q_0.5_ CB1073 = 1.09 mmol g^−1^). This behaviour can be correlated also with the physicochemical properties of the activated carbons and in particular with the values of pH_pzc_ (Table 2) and the amount of methylparaben with a negative charge in solution (Table 6). In all the cases, the activated carbons have a net positive surface charge, while methylparaben is almost neutral, this favouring the formation of different kind of interactions involving phenol groups present in methylparaben structure: in strong acidic conditions, donor–acceptor interactions between the aromatic ring of phenol (electron acceptor) and the surface carboxylic groups of the activated carbon (electron donor) and, in all pH conditions, Brønsted–Lowry acid-basic interactions with π electrons/basic groups and π-stacking interactions between the electrons in the aromatic rings of the methylparaben and those in basal planes of the carbonaceous structure. However, it is worth observing that the major role played by microporosity suggests that the π- interactions are largely predominant due to the greater potential energy generated in micropores.

Moreover, the possible formation of hydrogen bonds with acidic group (e.g., carboxylics) can be limited by the possible competition of water, endorsing their detrimental effect [19].

Hence, the functional groups present on carbon surface might also play a role to refine the analysis of the effect of the different chemical properties of the adsorbent, in the different investigated solvents.

**Figure 7:**
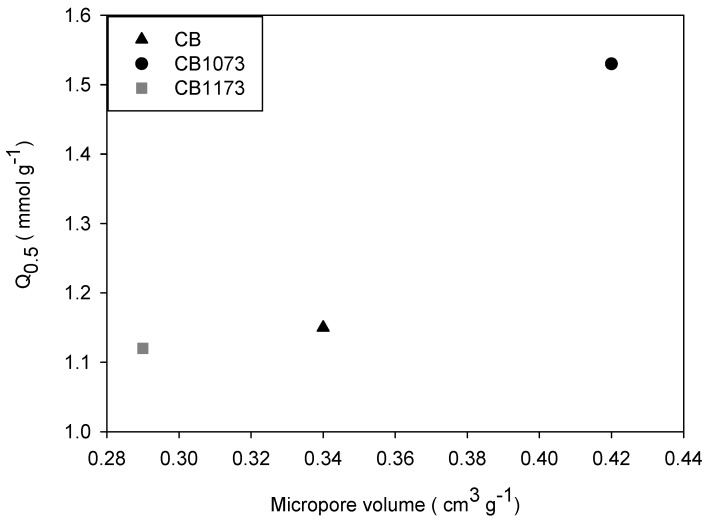
Relationship between the adsorbed amount of methylparaben in water (pH 7.75 ± 0.07) at 293 K and micropore volume on CB, CB1073 and CB1173 activated carbons.

For the adsorption of methylparaben on activated carbons in water (Figure 4), it was found that the methylparaben adsorbed amount (Q_0.5_) is higher for CB1073 (Q_0.5_ CB1073 = 1.53 mmol g^−1^) than CB (Q_0.5_ CB = 1.15 mmol g^−1^) and CB1173 (Q_0.5_ CB1073 = 1.12 mmol g^−1^). Figure 7 shows that the amount adsorbed (Q_0.5_) increases with the micropore volume of the activated carbons, as already observed.

According to Terzyk [34], the adsorption capacity of aromatic compounds depends on the formation of electrostatic interactions with functional groups located at the entrances of the micropores, which is known as primary micropore filling. Moreover, the Freundlich model assumes that the surface of the adsorbent is heterogeneous due to the presence of independent regions with different adsorption energy [35]. Hence, the moderate levels of heterogeneity (*n* = 0.45) for methylparaben adsorption in water (Table 5) suggest that adsorption occurs by micropores filling (for which the energy is the highest) and it depends also on the density of surface chemical groups and on the size of the pores that constitute a region (active sites). In fact, CB1073, which is the best performing activated carbon, is also characterized by the narrowest pore size distribution.

It can be also observed that for CB samples the methylparaben adsorption capacity is slightly lower than the value expected on the basis of its micropore volume. Indeed, a lower total basic group content (742 μmol g^−1^ for CB and 2037 μmol g^−1^ for CB1173), a higher phenol group content (46.6 μmol g^−1^ for CB and 6.36 μmol g^−1^ for CB1173) and a lower pH_pzc_ (5.40 for CB and 8.90 for CB1173) represent factors affecting its performance. For CB1073, the basic group content was average but a significantly higher micropore volume likely determined its best performances.

**Figure 8:**
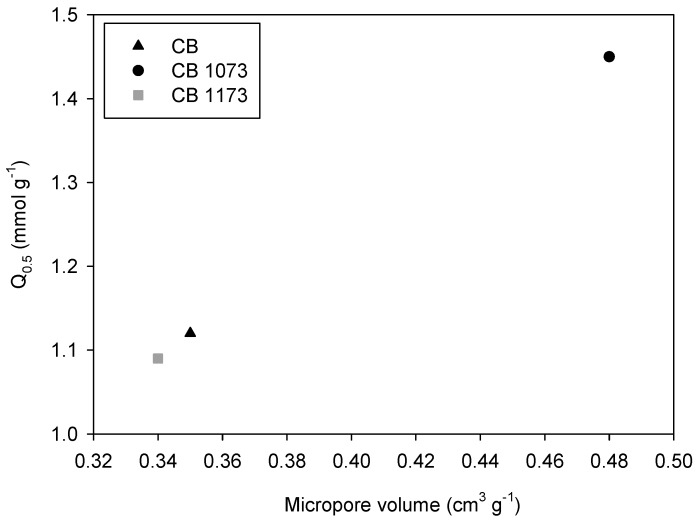
Relationship between the adsorbed amount of methylparaben in HCl 1 × 10^−2^ M at 293 K and micropore volume on CB, CB1073 and CB1173 activated carbons.

For adsorption data at acid pH values, the correlation between the amount adsorbed of methylparaben and micropore volume is confirmed with a more linear trend (Figure 8).

However, adsorption capacities at neutral pH are higher than those determined at acidic pH. In acidic conditions, the H^+^ excess determines the possible occurrence of hydrogen bonds between activated carbon and methylparaben molecules and a reduction of π-π interactions because of the diminution of the electron density of the basal planes of the activated carbon.

These two effects are likely to compensate for each other, the latter exerting a slightly greater influence on the overall slight reduction of adsorption capacity. In addition, water molecules can compete with methylparaben during adsorption, giving rise to adsorbed clusters and surface complexes, which can reduce its accessibility. Hence, it is confirmed that the π–π interactions, which are reduced in acidic conditions, are the main responsible of methylparaben adsorption capacity, as already observed.

For the adsorption of methylparaben in neutral/basic pH (Figure 5), similar observations can be derived as for data at neutral pH, mainly because the final pH are similar, and the micropore volume seems to be the main factor controlling the methylparaben adsorption performances. In detail, it can be observed that, again, CB1073 is favoured, being the methylparaben adsorbed amount (Q_0.5_ CB1073 = 1.48 mmol g^−1^) higher than CB (Q_0.5_ CB = 1.16 mmol g^−1^) and CB1173 (Q_0.5_ CB1173 = 1.14 mmol g^−1^). Compared with the test at acid pH, a slight increase in the adsorbed amount of methylparaben is observed, which is attributed to the reduction of water competition (as already observed for neutral pH) and, for CB1073, to the formation of additional electrostatic interactions between the negatively charged adsorbate molecules and the positive surface charge of activated carbon. On the other hand, CB and CB 1173 activated carbons determine repulsive forces with methylparaben due to their negative surface charge.

At high pH values the carboxylic acids formed are deprotonated and the negative charge generates repulsive effects with the ionized methylparaben molecules. However, due to acid-base reactions between the acid groups on the activated carbon and the hydroxides, the pH of the solution decreased and the percentage of negatively charged methylparaben molecules decreased too. In addition, the surface charge on CB1173 activated carbon is modified to positive and the Lewis acid-base, proton donor-acceptor and π-stacking interactions increased. For CB, the surface charge remains negative throughout the process, hence the adsorption of methylparaben onto this activated carbon is less influenced by surface chemistry and more by the interaction energy determined by micropores.

From adsorption data at different ionic strength levels, it can be observed that the adsorbed amount (Q_0.5_) is significantly affected by changes in the ionic strength, much more than by changes in the pH. Li et al. [32] indicate that the adsorption capacity of phenol increases with ionic strength; however, the results presented in Figure 6 (and Table 5) show an opposite trend. The highest adsorbed amount was reached for CB1073 and CB1173 activated carbons (Q_0.5_ CB1073 = 0.57 mmol g^−1^ and Q_0.5_ CB1173 = 0.56 mmol g^−1^, almost similar) while slightly lower performances were observed for CB (Q_0.5_ CB = 0.50 mmol g^−1^), the reduction being equal to 63%, 52% and 55% with respect to the homologous data in water.

The decrease in the methylparaben adsorbed amount on all the investigated activated carbons at high ionic strength is attributed to the competitive adsorption of Na^+^ and Cl^-^ ions in the micropores [36]; therefore, the methylparaben molecules can only interact with a reduced fraction of the basal plane of the activated carbons and with some of the surface groups present on the adsorbents. Moreover, the contribution of the π electron donor–acceptor interactions may be affected by the formation of interactions with Na^+^ and Cl^−^ ions in the medium, and therefore methylparaben must compete for the active sites, which have a greater affinity for ions due to the polarity and the stabilization of surface charges on the activated carbon. In this case, the trend of adsorption capacity does not strictly follow the one of micropore volume; in particular, CB1173 shows a higher adsorption capacity than CB but a lower micropore volume (0.29 cm^3^ g^−1^ vs. 0.34 cm^3^ g^−1^). This seems likely to be ascribed to the relation between equilibrium pH, almost neutral for both the tests (Table 5), and pH_PZC_, equal to 5.40 and 8.90 for CB and CB1173 (Table 2), respectively. Hence, in these conditions CB is negatively charged and the adsorption of Na^+^ is more favoured than on CB1173, which is positively charged.

### 2.3. Calorimetric Experiments

Adsorption data can be evaluated and better interpreted also by correlating the equilibrium adsorption results (i.e., adsorption isotherms) and the calorimetric data deriving from immersion calorimetry tests, for the quantification of the adsorbate–adsorbent–solvent interactions. In fact, one of the most reliable methods to determine the energies derived from the solvent–adsorbent and adsorbent–adsorbate interactions is the immersion calorimetry. This technique allows to measure the magnitude of the transferred energy when the activated carbon is put in contact with a pure liquid or a solution of a given compound and to correlate these data with the adsorbed amount of that compound. Table 7 shows the immersion enthalpy (*∆Himm _solvent_*) of CB, CB1073 and CB1173 activated carbons in the solvents used in the study, without methylparaben.

It is observed that the activated carbons subjected to thermal treatments (CB1073 and CB1173) have a slightly greater immersion enthalpy in acid medium due to the interaction between the basic groups, which are more abundant (Cf. Table 2), and the H^+^ ions. In basic medium, CB presents higher enthalpy values than CB1073 and CB1173 because it has lower basicity than the other activated carbons. The same trend found in the basic medium is observed for the immersion enthalpy in water, which might be associated also with a greater hydrophilicity due to the increase of the phenol group’s content on CB activated carbon surface.

The immersion enthalpies in NaCl solution show that, due to the basicity associated with the π electrons, the CB1073 and CB1173 activated carbons present a greater interaction with the Na^+^ ions than CB sample. The highest immersion enthalpy in NaCl solution was determined for CB1073, so it is confirmed that Na^+^ and Cl^−^ ions are adsorbed in the micropores as this activated carbon has the highest micropore volume among the samples tested in this study.

Table 8 shows the immersion enthalpies (*ΔHimm _MP_*) of CB, CB1073 and CB1173 activated carbons in methylparaben solutions at different concentrations and in all the investigated solvents (i.e., water, HCl, NaOH and NaCl solutions). In Table 8, the letter L represents the tests made at low methylparaben concentration (ranging between 0.07–0.66 mmol L^−1^) while and the letter H represents those made at high methylparaben concentration (ranging between 1.31–6.58 mmol L^−1^).

In Table 8, it is observed that the absolute values of the immersion enthalpy in the methylparaben solutions decrease with respect to the values determined for the same pure solvents, keeping their exothermic character. This indicates that the presence of the solute decreases the magnitude of solvent–adsorbent interactions and/or determines the occurrence of more complex phenomena in the solution, as discussed below. This trend has some exceptions at low methylparaben concentration, in particular for CB1073 activated carbon in neutral pH, for which the interaction with the solvent has been already demonstrated to be weaker. Likewise, for CB1073 activated carbon, the immersion enthalpy increases at low methylparaben concentration in NaCl solutions because the interactions of the π electrons of the adsorbate with Na^+^ and Cl^−^ ions in the solvent increase. As expected, for all the activated carbons, an increase in immersion enthalpy values is observed when methylparaben concentration is higher, indicating an increase in adsorbate–adsorbent interactions due to the occupation of more active sites by the adsorbate. The enthalpy values determined for CB1073 in acid medium (−98.0 J g^−1^) and NaCl (−96.1 J g^−1^) indicate the simultaneous interactions between methylparaben, H^+^ and Na^+^ Cl^−^ ions with the activated carbon surface, as indicated in the analysis of the equilibrium data.

In order to evaluate the magnitude of the specific interactions methylparaben-activated carbon, the interaction enthalpy (*ΔH_int MP_*_−*AC*_) can be determined. This was made in correspondence of the highest methylparaben concentration (i.e., 6.58 mmol L^−1^, cf. Table 8), because the calorimetric effect is more evident and, consequently, the retrieved results more reliable. The change in the interaction enthalpy can show either positive or negative values. In the first case, the process is endothermic and it requires energy to be carried out, hence it is usually associated with the breaking of chemical bonds or interactions. On contrary, negative enthalpies correspond to an exothermic process, which releases energy due to the formation of new adsorbent–adsorbate interactions.

**Figure 9:**
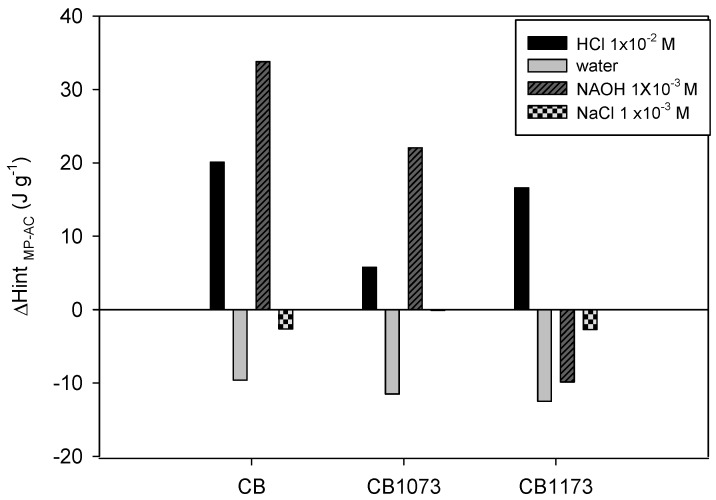
Changes in the interaction enthalpy of methylparaben (*ΔH_int MP_*_−_*_AC_*) and CB, CB1073 and CB1173 activated carbons activated carbons in different solvents (i.e., HCl, water NaOH and NaCl) at 6.58 mmol L^−1^ methylparaben concentration.

In Figure 9, the interaction enthalpy values of the different activated carbons and in the different solutions are reported, assuming that they are mainly derived from the interaction of methylparaben with carbon basal plane (i.e., π interactions) and surface functional groups (i.e., carboxylic acids).

The retrieved values of *ΔHint _MP−AC_* confirm that methylparaben-AC interactions depend on pH and ionic strength. In water, it is observed that negative values of enthalpy increased (absolute value) slightly with the basicity of the activated carbons, which is associated with the formation of interactions by π- electrons and basic functional groups. These interactions are disadvantaged by pH and ionic strength changes due to the occurrence of interactions between the cation (H^+^ Na^+^) - π electron of the activated carbon and the same ions in the medium and the aromatic ring of the adsorbate. Consequently, for experiments carried out in HCl and NaOH, an endothermic contribution ascribable to the de-solvatation of methylparaben molecules and solvent displacement from activated carbon surface arises and, differently than what expected, cannot be neglected. The importance of π-stacking interactions is visible at basic pH where the activated carbon with greater basicity (CB1173) presents an exception to this trend, with a negative change in the enthalpy. Additionally, it is confirmed that the ionic interactions are not relevant in the formation of methylparaben–AC interactions, because when the adsorbate is negatively charged (98% ionized) and interactions with CB1073 activated carbon (positively charged) should be favoured, the change in enthalpy is positive (22.05 J g^−1^).

Finally, it is confirmed that the presence of Na^+^ Cl^−^ ions in the medium interferes with the formation of methylparaben-AC interactions since the interaction enthalpy values are closed to zero.

In order to determine the contribution of the ester group in the adsorbate-adsorbent interactions, the interaction enthalpy ester–AC was calculated starting from the interaction enthalpies of methylparaben–AC and phenol–AC (the latter previously determined in the same experimental conditions as those used for methylparaben tests). For this calculation, it is assumed that the enthalpy is an additive–constitutive property of the molecules (hence the Hess law can be applied), so that the interaction enthalpy of methylparaben–AC corresponds to the sum of the phenol and the ester interaction enthalpies with the activated carbons. In order to avoid the effect caused by pH on the different ionization of phenol and methylparaben molecules, which would not allow a fair comparison, only water was considered as a solvent. In fact, in this case, the phenol solution had a pH of 7.05 ± 0.21 and an ionization percentage of 11%, which are very similar values to those retrieved in methylparaben experiments in water (cf. Table 6).

**Figure 10:**
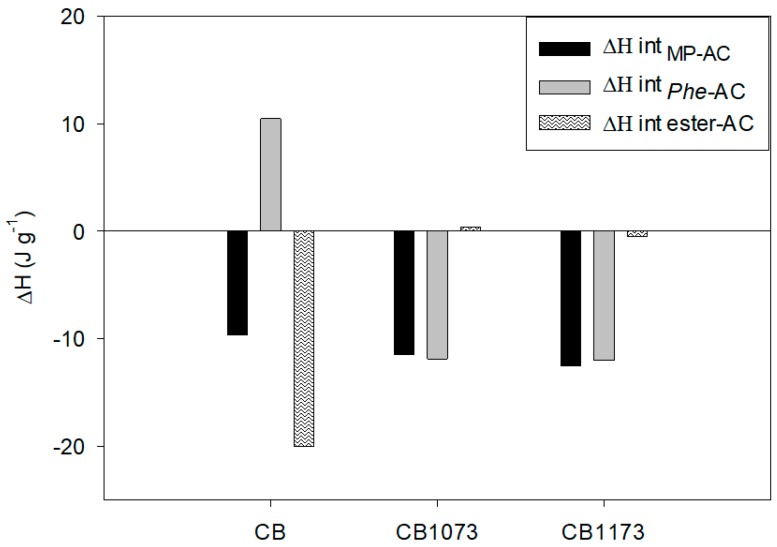
Enthalpic contribution derived from the ester and phenol group present in methylparaben structure in the adsorbate-adsorbent interactions with CB, CB 1073 and CB 1173 activated carbons in water.

The contributions of methylparaben–AC, phenol–AC and of the ester group in the interactions adsorbate–adsorbent in water are presented in Figure 10.

Figure 10 shows that the contribution of the ester group in the interaction enthalpy methylparaben–AC is greater for the CB activated carbon and almost zero for CB1073 and CB1173 samples. This behaviour is likely related to the surface chemistry of the activated carbons, since CB activated carbon has a greater amount of phenol groups (46.6 µmol g^−1^) and a lower amount of carboxylic acids (22.2 µmol g^−1^) which allow the formation of Brønsted–Lowry acid–base interactions involving the ester (proton acceptor) and phenol groups of activated carbon (proton donor). It is interesting to observe that for the CB sample an endothermic effect in phenol–AC interactions, likely associated with the solvatation of phenol groups both in solution and on carbon surface, is present. This behaviour is reversed on CB1073 and CB1173 activated carbons, since for these adsorbents the contribution of π-electron interactions between carbon basal plane and the aromatic group of methylparaben molecule are largely predominant, thus determining *ΔHint _MP−AC_* ≈ *ΔHint _Phe−AC_* (i.e., the ester group of methylparaben has negligible influence on its adsorption on these sorbents).

## 3. Materials and Methods

### 3.1. Activated Carbons Characterization

Three activated carbons with different physicochemical characteristics were used as adsorbents. CB is a commercial activated carbon (Carbochem Brand GS50, Carbochem Inc., Philadelphia, PA, USA) prepared from coconut shell. Table 9 summarizes the technical characteristics of CB activated carbon. Before the use, the activated carbon was washed with a diluted HCl solution in order to eliminate the inorganic impurities and then was washed with distilled water to remove the excess of HCl; finally, it was dried at 373 K. Other physico-chemical characteristics of CB activated carbon are reported by Rodriguez-Estupiñan [37].

CB1073 and CB1173 activated carbons were obtained from CB sample through a same thermal treatment but carried out at different temperatures, i.e., 1073 and 1173 K, respectively. To this aim, 100 g of CB activated carbon were placed in a THERMOLYNE furnace (Thermo Fisher Scientific, Madison, WI, USA) and left for 2 h at the assigned temperature with a 2 K s^−1^ heating ramp in nitrogen atmosphere. Afterwards, the activated carbon was cooled in the furnace and stored in amber glass jars with an airtight seal.

#### 3.1.1. Physical Characterization 

The textural properties of the activated carbons were determined from N_2_ adsorption isotherms carried out at 77 K in a commercial semi-automatic autorsorb IQ2 sorptometer (Quantachrome Instruments, Boynton Beach, FL, USA), after degassing the activated carbons for 24 h at 473 K.

The BET, DA and QSDFT models were applied to nitrogen isotherms for the determination of the apparent surface area, micropore volume and pore size distribution, respectively.

#### 3.1.2. Chemical Characterization

The determination of the acidic surface chemical groups (phenol, lactone and carboxylic) was made by back titration with bases having a different strength, which allow to neutralize acids with different pKa and then determine their single contributions [38]. To this aim, 0.5 g of activated carbon (either CB, CB 1073 or CB 1173) was weighted on an analytical balance with an accuracy of 0.001 g (Ohaus Pioneer PA 114, Ohaus Corporation, Parsippany, NJ, USA) and then put in hermetic glass containers. Subsequently, 0.05 L of a 0.1 M solution of NaOH was added and kept at room temperature (293 K ± 1 K) for 5 days and under 100 rpm constant agitation. After, an aliquot of the supernatant was sampled and titrated with HCl using a potentiometer (CG840 Schoot, Schott AG, Maguncia, Germany). The same procedure was repeated using sodium carbonate (Na_2_CO_3_) and sodium bicarbonate (NaHCO_3_) as immersion liquids so to obtain a complete quantification of the acidic functional groups. For the quantification of basic groups, the same procedure was adopted, using 0.1 M solution of HCl as the immersion liquid.

The HCl and NaOH solutions were previously standardized with boric acid and potassium biphthalate.

Some of the properties associated with the adsorption in solid–liquid systems are influenced by the appearance of surface electric charges [39]. When activated carbon is placed in contact with an electrolytic solution, the surface ionizes depending on the pKa of the functional groups present on the surface, and the charged particle is surrounded by ions of opposite charge, so that it is electrically neutral. The pH value required for the net surface charge of the activated carbon to be zero is known as the pH at the point of zero charge (pH_pzc_). For its determination, a variable amount of 0.05–0.5 g of activated carbon (CB, CB 1073 or CB 1173) was weighted and then put in amber glass containers with a lid. Thereafter, 0.01 L of a 0.1 M NaCl solution was added. The containers were stored for 5 days at room temperature (293 ± 1 K) and constant agitation (100 rpm); after the equilibrium time, 0.005 L of the supernatant solution of each container is taken and the pH is measured with a CG 840 Schott equipment (Germany).

### 3.2. Batch Adsorption Tests

The stock solution of methylparaben was prepared by weighing 2 g of methylparaben with a purity of 99% (Panreac chemistry SLU, Castellar del Vallès, Barcelona, Spain) and then added to 1 L of working solution, stirred at 150 rpm and stored at 293 K in an amber bottle. The working solutions were prepared in distilled water, HCl, NaOH and NaCl were used for the tests carried out at different pH or ionic strength. The solutions of NaOH, HCl and NaCl were prepared with distilled water and analyte grade reagents between 97 and 99% purity (Merck Millipore, Bedford, MA, USA). A constant amount of 0.1 ± 0.001 g of activated carbon (CB, CB 1073, CB 1173) was put in amber glass containers and 0.025 L of the methylparaben solution with concentration ranging between 0.07–6.58 mmol L^−1^ was added. The containers were stored for 10 days at room temperature (293 ± 1 K) and with a sporadic agitation (100 rpm).

After equilibration, the solutions were filtered, an aliquot was taken and the adsorbate concentration was determined by ultraviolet-visible (UV-vis) spectroscopy (GENESYS 10S Vis spectrophotometer, Thermo Fisher Scientific, Madison, WI, USA). The maximum wavelengths (λ) for methylparaben determination are shown in Table 10.

The methylparaben-adsorbed amount on the activated carbon was calculated through a material balance, as reported in Equation (2)

(2) $$Q=\frac{\left(C_{o}-C_{e}\right)V}{m}$$

where *Q* is the adsorbed amount, *C_o_* is the initial concentration, *C_e_* is the equilibrium concentration, *V* the total volume and *m* is the mass of the activated carbon.

### 3.3. Immersion Calorimetry

The immersion enthalpies of CB, CB1073 and CB1173 activated carbons were determined in methylparaben solutions with concentrations ranging between 0.07 and 6.58 mmol L^−1^ using different solvents, namely distilled water or HCl, NaOH and NaCl solutions at 293 ± 1 K. Additionally, immersion calorimetry runs were carried out in the same pure solvents and in phenol solutions.

The measurements were made in a Calvet heat conduction microcalorimeter. To this aim, 0.1 ± 0.01 g of activated carbon was put in a glass ampoule, which is placed on the top cover of the equipment. Previously, 0.01 L of the immersion solution was deposited inside the calorimeter in a dedicated stainless steel cell. Once the calorimeter is closed, the electric potential signal was acquired for approximately 1 h to get a stable baseline; thereafter, the glass ampoule was broken and the potential peak due to the immersion was recorded until it back again to the base line and the electrical calibration can be performed.

The immersion enthalpy was calculated from Equations (3)–(6), where *W_ET_* corresponds to the electrical work from electrical calibration, *K_cal_* is the constant of the calorimeter, *Q_imm_* is the heat of immersion and *ΔH_imm_* is the immersion enthalpy. It can be observed that the change in enthalpy depends on the area of electric potential peaks generated upon immersion and with calibration, as well as the calorimetric constant calculated from electrical calibration.

(3) $$W_{ET}=voltage\;\left(V\right)*current\;\left(A\right)*time\;(s)$$

(4) $$K_{cal}=\frac{W_{ET}}{Area\;under\;curve\;\left(calibration\;peak\right)}$$

(5) $$Q_{imm}=K_{cal}*\;Area\;under\;curve\;\left(immersion\;peak\right)\;$$

(6) $$\Delta H_{imm}=\frac{Q_{imm}\;\left(J\right)}{activated\;carbon\;mass\;\left(g\right)}$$

The methylparaben interaction enthalpy (*ΔH_int MP−AC_*) was determined from the application of the Hess law [40], assuming that the solutions are infinitely diluted. It is also assumed that the endothermic effects derived from water displacement from the carbonaceous surface and the de-solvatation of the solute before adsorption are accounted for in the potential curve originated during the immersion. Therefore, the interaction enthalpy depends not only on the adsorbent–adsorbent (exothermic) interactions but also on the breakdown of adsorbate–solvent and solvent–adsorbent interactions. Equation (7) shows the mathematical expression used to determine the enthalpy of the interaction in this study.

(7) $$\Delta Hint_{MP-AC}=\left(\Delta Himm_{MP}\right)-\left(\Delta Himm_{solvent}\right)$$

in which *ΔHimm_MP_* represents the immersion enthalpy in 6.58 mmol L^−1^ of methylparaben solutions at and the *ΔHimm_solvent_* represents the immersion enthalpy in the solvent (i.e, water, HCl, NaOH, NaCl) without methylparaben.

The effect of the ester group in methylparaben molecule on the formation of adsorbate–adsorbent interactions was determined using the Hess law, as shown in Equation (8).

(8) $$\Delta Hint_{ester-AC}=\left(\Delta Hint_{MP-AC}\right)-\left(\Delta Hint_{Phe-AC}\;\right)$$

where *ΔHint_ester−AC_* represents the enthalpy change associated to the interaction ester-activated carbon, *ΔHint_MP−AC_* represents the methylparaben-activated carbon interaction enthalpy in solutions (at 6.58 mmol L^−1^) and *ΔHimm_Phe−AC_* is the is the interaction enthalpy determined for a phenol solution under same test conditions used with methylparaben.

## 4. Conclusions

This work investigated the adsorption of methylparaben on 3 different activated carbons, obtained by thermal treatments of a same raw sample (CB) in different pH and ionic strength aqueous solutions.

The thermal treatments at 1073 and 1173 K determined an increase in the basicity of the activated carbons (CB1073 and CB1173). However, only the thermal treatment at 1073 K allowed a significant increase of the apparent surface area and the micropore volume with respect to the raw sample and, for these reasons, methylparaben adsorption was favoured on CB1073. For all the investigated activated carbons, the ranking of adsorption capacity between the different solution pH resulted in the same H_2_O ≅ NaOH > HCl, even if the differences were small, while adsorption capacity in NaCl solution was the lowest by far, because it was reduced by the formation of cation (H^+^ Na^+^) - π electron interactions between aromatic ring of methylparaben and the solvent. The analysis of the effect of pH on methylparaben ionization in solution indicated that this phenomenon exerts a slight effect on adsorption capacity, only if correlated to the pH_pzc_ of the adsorbents. In fact, methylparaben adsorption is disfavoured for pH value that determines a simultaneous negative charge of the adsorbent (pH > pH_pzc_) and high ionization degree of the adsorbate (pH > pKa). A finer analysis of the experimental results confirmed that π-stacking interactions are largely predominant and mainly promoted by a high micropores volume, while the possible detrimental effect exerted by the acidic group is slight.

The formation of interactions by π- stacking between activated carbon graphitic layer and methylparaben molecule were confirmed to play an important role in methylparaben adsorption. These interactions are disadvantaged by pH and ionic strength changes due to the occurrence of interactions between cation (H^+^ Na^+^) - π electron and anion (OH^−^ Cl^−^) - π electrons. Consequently, an endothermic contribution ascribable to the de-solvatation of methylparaben molecules and solvent–adsorbate displacement on activated carbon surface was detected. The change in ionic strength disadvantage the methylparaben adsorption due to the preferential adsorption of Na^+^ and Cl^−^ ions on activated carbon, which is confirmed by the interaction enthalpies of methylparaben-AC close to zero.

Finally, calorimetric data results showed that the ester group of methylparaben contributes to the formation of adsorbate–adsorbent interactions only on activated carbons with a higher content of proton-donor chemical groups such as phenols (CB sample) since it is involved in the formation of acid-base interactions.

## Figures and Tables

**Table 1:** Main physical characteristics of CB, CB1073 and CB1173 activated carbons.

	CB	CB 1073	CB 1173
Surface area (S_BET_) (m^2^ g^−1^)	864	1127	814
Micropore volume (cm^3^ g^−1^) (V_m_ − DA)	0.34 *n* = 2.1	0.42 *n* = 2.1	0.29 *n* = 2.3
Total volume (cm^3^ g^−1^) (V_T_)	0.3	0.48	0.34

**Table 2:** Chemical characteristics of CB, CB1073 and CB1173 activated carbons.

	CB	CB1073	CB1173
Total acidity (µmol g^−1^)	90.5	93.6	93.0
Total basicity (µmol g^−1^)	742	1210	2037
Carboxylic acids (µmol g^−1^)	22.2	66.1	65.5
Lactones (µmol g^−1^)	21.8	21.2	23.8
Phenols (µmol g^−1^)	46.6	6.36	3.71
pH_pzc_	5.40	11.1	8.90

**Table 3:** Physicochemical properties of methylparaben in different solutions [30].

Methylparaben				
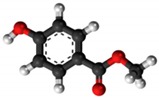 Black spheres: carbon atoms; white spheres: hydrogen atoms; red spheres: oxygen atoms.				
Molecular Dimensions (nm^2^) 0.87 × 0.50		Molecular Weight 152 g mol^−1^		
Range of Concentrations: 0.07mmol L^−1^ to 6.58 mmol L^−1^				
	H_2_O	HCl solution 1 × 10^−2^ M	NaOH solution 1 × 10^−3^ M	NaCl Solution 1 × 10^−2^ M
Log Solubility (298 K)	−1.29	−1.29	0.71	Und ^b^
pKa ^a^	8.20	NA ^c^	NA	8.25

^a^ Reported by Pubchem ^b^ Undetermined ^c^ Not Applicable.

**Table 4:** Freundlich model parameters for methylparaben adsorption on CB, CB1073 and CB1173 activated carbons in HCl 1 × 10^−2^ M, water, NaOH 1 × 10^−3^ M and NaCl 1 × 10^−2^ M solutions at 293 K.

		K	n	R^2^
HCl 1 × 10^−2^ M	CB	1.38	0.30	0.96
	CB-1073	1.90	0.39	0.98
	CB-1173	1.40	0.36	0.94
Water	CB	1.57	0.45	0.96
	CB-1073	2.08	0.44	0.97
	CB-1173	1.53	0.45	0.96
NaOH 1 × 10^-3^ M	CB	1.45	0.32	0.94
	CB-1073	1.94	0.39	0.95
	CB-1173	1.44	0.34	0.95
NaCl 1 × 10^−2^ M	CB	0.63	0.32	0.96
	CB-1073	0.74	0.37	0.96
	CB-1173	0.72	0.37	0.97

**Table 5:** Methylparaben adsorption capacities of CB, CB1073 and CB1173 activated carbons in different solutions, calculated by Freundlich model.

	Q_0.5_ HCl Solution (mmolg^−1^)	Q_0.5_ H_2_O (mmol g^−1^)	Q_0.5_ NaOH Solution (mmol g^−1^)	Q_0.5_ NaCl Solution (mmol g^−1^)
CB	1.12	1.15	1.16	0.50
CB 1073	1.45	1.53	1.48	0.57
CB 1173	1.09	1.12	1.14	0.56

**Table 6:** pH values and percentage of ionization of the methylparaben in different solutions, before and after adsorption on CB, CB1073 and CB1173 activated carbons.

Solvent	Sample	Initial pH	% Ionization	Final pH	% Ionization
HCl 1 × 10^−2^ M	CB	1.56 ± 0.03	0.00	1.2 ± 0.02	5.94
	CB 1073			1.11 ± 0.02	7.20
	CB 1173			1.24 ± 0.02	5.44
Water	CB	6.57 ± 0.13	0.17	7.54 ± 0.15	18.1
	CB 1073			7.83 ± 0.16	29.9
	CB 1173			7.88 ± 0.16	32.4
NaOH 1 × 10^−3^ M	CB	9.80 ± 0.19	98.0	7.68 ± 0.15	23.2
	CB 1073			10.0 ± 0.20	98.4
	CB 1173			7.95 ± 0.16	36.0
NaCl 1 × 10^−2^ M	CB	7.02 ± 0.14	5.60	7.13 ± 0.15	7.05
	CB 1073			7.14 ± 0.15	7.20
	CB 1173			7.86 ± 0.15	28.9

**Table 7:** Immersion enthalpies (*ΔHimm _solvent_*) of CB, CB1073 and CB1173 activated carbons in different solvents (water, HCl, NaOH and NaCl solutions) without methylparaben.

	−ΔH HCl 1 × 10^−2^ M (J g^−1^)	−ΔH Water (J g^−1^)	−ΔH NaOH 1 × 10^−3^ M (J g^−1^)	−ΔH NaCl 1 × 10^−^^2^ M (J g^−^^1^)
CB	51.4 ± 1.02	49.7 ± 0.99	57.3 ± 1.15	24.3 ± 0.49
CB1073	56.2 ± 1.12	27.4 ± 0.55	49.4 ± 0.99	39.2 ± 0.78
CB1173	58.2 ± 1.16	32.4 ± 0.65	31.5 ± 0.63	36.0 ± 0.72

**Table 8:** Immersion enthalpies (*∆Himm* *_MP_*) of CB, CB1073 and CB1173 activated carbons in different solvents (i.e., water, HCl, NaOH and NaCl) at two different range of methylparaben concentration.

	ΔH HCl 1 × 10^−2^ M (J g^−1^)		ΔH Water (J g^−1^)		ΔH NaOH 1×10^−3^ M (J g^−1^)		ΔH NaCl 1×10^−^^2^ M (J g^−^^1^)	
	L	H	L	H	L	H	L	H
CB	−17.4	−31.0	−11.9	−51.0	−13.9	−26.0	−10.7	−31.7
CB1073	−49.6	−98.0	−41.2	−42.6	−18.6	−34.3	−63.4	−96.1
CB 1173	−15.5	−47.8	−11.7	−37.1	−9.26	−42.0	−28.9	−38.6

N.B. The immersion enthalpy values in methylparaben solutions have error percentages lower than 2%.

**Table 9:** Datasheet of raw activated carbon (CB).

Precursor	Coconut Shell
Type of activation	physical activation with CO_2_
pH_pzc_	5.00–7.00
Iodine number	850–950 mg I g^−1^
Density	450–500 g L^−1^
Particle size	1.00 mm

**Table 10:** Maximum wavelengths of methylparaben in different solvents.

	λ H_2_O (nm)	λ HCl 1 × 10^−2^ M (nm)	λ NaOH 1 × 10^−3^ M (nm)	λ NaCl 1 × 10^−2^ M (nm)
Methylparaben	254	254	294	196

## References

[1] Chen H.W., Chiou C.S., Chang S.H. (2017). Comparison of methylparaben, ethylparaben and propylparaben adsorption onto magnetic nanoparticles with phenyl group. Powder Technol..

[2] Giulivo M., de Alda M.L., Capri E., Barceló D. (2016). Human exposure to endocrine disrupting compounds: Their role in reproductive systems, metabolic syndrome and breast cancer. A review. Environ. Res..

[3] Spindola Vilela C., Bassin J., Silva Peixoto R. (2018). Water contamination by endocrine disruptors: Impacts, microbiological aspects and trends for environmental protection. Environ. Pollut..

[4] Zgoła-Grześkowiak A., Jeszka-Skowron M., Czarczyńska-Goślińska B., Grześkowiak T. (2016). Determination of parabens in polish river and lake water as a function of season. Anal. Lett..

[5] World Health Organization (2012). Pharmaceuticals in Drinking-Water.

[6] Sharma A., Ahmad J., Flora S.J.S. (2018). Application of advanced oxidation processes and toxicity assessment of transformation products. Environ. Res..

[7] Gomaa H., Khalifa H., Selim M.M., Shenashen M.A., Kawada S., Alamoudi A.S., El-Safty S.A. (2017). Selective, photoenhanced trapping/detrapping of arsenate anions using mesoporous blobfish head TiO_2_ monoliths. ACS Sustain. Chem. Eng..

[8] Canosa P., Rodríguez I., Rubi E., Negreira N., Cela R. (2006). Formation of halogenated by-products of parabens in chlorinated water. Anal. Chim. Acta.

[9] Wu Y., Sun Q., Wang Y.W., Deng C.X., Yu C.P. (2017). Comparative studies of aerobic and anaerobic biodegradation of methylparaben and propylparaben in activated sludge. Ecotoxicol. Environ. Saf..

[10] Yoom H., Shin J., Ra J., Son H., Ryu D., Kim C., Lee Y. (2018). Transformation of methylparaben during water chlorination: Effects of bromide and dissolved organic matter on reaction kinetics and transformation pathways. Sci. Total. Environ..

[11] De Oliveira T., Guégan R., Thiebault T., Le Milbeau C., Muller F., Teixeira V., Boussafir M. (2017). Adsorption of diclofenac onto organoclays: Effects of surfactant and environmental (pH and temperature) conditions. J. Hazard Mater..

[12] Garcia-Ivars J., Durá-María J., Moscardó-Carreño C., Carbonell-Alcaina C., Alcaina-Miranda M.I., Iborra-Clar M.I. (2017). Rejection of trace pharmaceutically active compounds present in municipal wastewaters using ceramic fine ultrafiltration membranes: Effect of feed solution pH and fouling phenomena. Sep. Purif. Technol..

[13] Yang Y., Hu X., Zhao Y., Cui L., Huang Z., Long J., Liao W. (2017). Decontamination of tetracycline by thiourea-dioxide–reduced magnetic graphene oxide: Effects of pH, ionic strength, and humic acid concentration. J. Colloid Interf. Sci..

[14] Nayak A., Bhushan B., Gupta V., Sharma P. (2017). Chemically activated carbon from lignocellulosic wastes for heavy metal wastewater remediation: Effect of activation conditions. J. Colloid Interf. Sci..

[15] Kwiatkowski M., Kalderis D., Diamadopoulos E. (2017). Numerical analysis of the influence of the impregnation ratio on the microporous structure formation of activated carbons, prepared by chemical activation of waste biomass with phosphoric (V) acid. J. Phys. Chem. Solids.

[16] Sun K., Leng C.Y., Jiang J.C., Bu Q., Lin G.F., Lu X.C., Zhu G.Z. (2018). Microporous activated carbons from coconut shells produced by self-activation using the pyrolysis gases produced from them, that have an excellent electric double layer performance. Carbon.

[17] Dąbrowski A., Podkościelny P., Hubicki Z., Barczak M. (2005). Adsorption of phenolic compounds by activated carbon—A critical review. Chemosphere.

[18] Mallek M., Chtourou M., Portillo M., Monclús H., Walha K., ben Salah A., Salvadó V. (2018). Granulated cork as biosorbent for the removal of phenol derivatives and emerging contaminants. J. Environ. Manag..

[19] Vargas D.P., Giraldo L., Moreno-Piraján J.C. (2017). Effect of textural and chemical characteristics of activated carbons on phenol adsorption in aqueous solutions. Pol. J. Chem. Technol..

[20] Ma D., Chen L., Liu R. (2017). Removal of novel antiandrogens identified in biological effluents of domestic wastewater by activated carbon. Sci. Total. Environ..

[21] Almeida C., Nogueira J.M.F. (2014). Determination of trace levels of parabens in real matrices by bar adsorptive microextraction using selective sorbent phases. J. Chromatogr. A.

[22] Román S., Ledesma B., Álvarez-Murillo A., Al-Kassir A., Yusaf T. (2017). Dependence of the microporosity of activated carbons on the lignocellulosic composition of the precursors. Energies.

[23] Carvajal-Bernal A.M., Gómez-Granados F., Giraldo L., Moreno-Piraján J.C. (2018). A study of the interactions of activated carbon-phenol in aqueous solution using the determination of immersion enthalpy. Appl. Sci..

[24] Marsh H., Reinoso F.R. (2006). Activated carbon.

[25] Avelino F., da Silva K.T., de Souza M.D.S.M., Mazzetto S.E., Lomonaco D. (2018). Microwave-assisted organosolv extraction of coconut shell lignin by Brønsted and Lewis acids catalysts. J. Clean Prod..

[26] De la Puente G., Pis J.J., Menéndez J.A., Grange P. (1997). Thermal stability of oxygenated functions in activated carbons. J. Anal. Appl. Pyrolysis..

[27] Rodríguez-Estupiñán P., Giraldo L., Moreno-Piraján J.C., Crini G., Lichtfouse E. (2018). Carbonaceous porous materials for the adsorption of heavy metals: Chemical characterization of oxidized activated carbons. Green Adsorbents for Pollutant Removal.

[28] da Silva W.L., Salomão A.A., Vila M.M., Tubino M. (2017). Influence of water and ultraviolet irradiation on the induction period of the oxidation of biodiesel. J. Braz. Chem. Soc..

[29] Wu H., Li Z., Liu H. (2018). Development of carbon adsorbents with high surface acidity and basicity from polyhydric alcohols with phosphoric acid activation for Ni (II) removal. Chemosphere.

[30] Giordano F., Bettini R., Donini C., Gazzaniga A., Caira M.R., Zhang G.G., Grant D.J. (1999). Physical properties of parabens and their mixtures: Solubility in water, thermal behavior, and crystal structures. J. Pharm. Sci..

[31] Melo L.P., Queiroz M.E.C. (2010). Simultaneous analysis of parabens in cosmetic products by stir bar sorptive extraction and liquid chromatography. J. Sep. Sci..

[32] Li J., Zhang K., Zhang H. (2018). Adsorption of antibiotics on microplastics. Environ. Pollut..

[33] Yang C.H. (1998). Statistical mechanical study on the Freundlich isotherm equation. J. Colloid Interf. Sci..

[34] Terzyk A.P. (2004). Molecular properties and intermolecular forces—factors balancing the effect of carbon surface chemistry in adsorption of organics from dilute aqueous solutions. J. Colloid Interf. Sci..

[35] Do D.D., Yang R. (1998). Practical approaches of pure component adsorption equilibria. Adsorption analysis: Equilibria and Kinetics.

[36] Zhao S., Yan T., Wang Z., Zhang J., Shi L., Zhang D. (2017). Removal of NaCl from saltwater solutions using micro/mesoporous carbon sheets derived from watermelon peel via deionization capacitors. RSC Adv..

[37] Rodríguez Estupiñan P., Giraldo L., Moreno-Piraján J.C. (2011). Oxidación de la superficie de carbón activado mediante HNO_3_ y H_2_O_2_: Efecto sobre la remoción de níquel (II) en solución acuosa. Rev. Colomb. Quim..

[38] Boehm H.P. (2002). Surface oxides on carbon and their analysis: A critical assessment. Carbon.

[39] Noh J.S., Schwarz J.A. (1989). Estimation of the point of zero charge of simple oxides by mass titration. J. Colloid Interf. Sci..

[40] Degaga G.D., Valenzano L. (2017). Part II: Quantum mechanical prediction of heats of adsorption for C2-C4 hydrocarbons in MOF-74-Mg/Zn periodic structures. Chem. Phys. Lett..

